# Combined central and peripheral nervous system involvement in acute disseminated encephalomyelitis in a young male: A case report

**DOI:** 10.1097/MD.0000000000048656

**Published:** 2026-05-01

**Authors:** Aasim Ali, Muhammad Shamoon, Muhammad Asad Shabbir, Fiza Nisar, Danish Yousuf, Gohar Mushtaq, Muhammad Abdullah Ali, Anousha Tanveer, Hammad Azam, Mukesh Kumar Sharma

**Affiliations:** a Neurology Department, Allied Hospital Faisalabad, Faisalabad, Pakistan; b Internal Medicine Department, Allied Hospital Faisalabad, Faisalabad, Pakistan; c Neurology Department, Tribhuvan University – Dhankuta Multiple Campus, Dhankuta, Nepal.

**Keywords:** acute disseminated encephalomyelitis, motor axonal neuropathy, MRI, peripheral neuropathy, plasma exchange, young adult

## Abstract

**Rationale:**

Acute disseminated encephalomyelitis (ADEM) is an autoimmune demyelinating disorder of the central nervous system, typically presenting after infection or vaccination. Rarely, ADEM may involve the peripheral nervous system, presenting with pure motor axonal neuropathy, which poses a diagnostic and therapeutic challenge.

**Patient concerns:**

We report a 22-year-old Asian male with no prior medical history who initially experienced a mild upper respiratory infection followed by headache and a single generalized tonic–clonic seizure. He subsequently developed altered consciousness and flaccid paraplegia.

**Diagnoses:**

Brain magnetic resonance imaging demonstrated asymmetric, poorly marginated supratentorial white-matter hyperintensities on T2-weighted and fluid-attenuated inversion recovery sequences. Cerebrospinal fluid analysis revealed mild lymphocytic pleocytosis and elevated protein, while autoimmune encephalitis panels and infectious studies were negative. Nerve conduction studies revealed predominantly motor axonal neuropathy affecting the lower limbs, with sensory sparing.

**Interventions:**

The patient received high-dose intravenous methylprednisolone followed by therapeutic plasma exchange due to incomplete motor recovery.

**Outcomes:**

After 2 weeks, he regained the ability to stand and ambulate with support, and repeat magnetic resonance imaging at 3 months demonstrated complete resolution of the lesions.

**Lessons:**

This case highlights a rare presentation of ADEM with peripheral nervous system involvement in a young adult, distinguished by predominant motor axonal neuropathy and a favorable response to plasma exchange. Recognition of peripheral involvement in ADEM is essential, particularly in younger patients with persistent weakness despite corticosteroid therapy, as timely diagnosis and intervention can significantly improve functional outcomes.

## 1. Introduction

Acute disseminated encephalomyelitis (ADEM) is an immune-mediated inflammatory disorder characterized by acute onset encephalopathy and multifocal neurological deficits, typically following an antecedent infection or, less commonly, vaccination. The hallmark radiological feature of ADEM is asymmetric, multifocal white matter lesions on magnetic resonance imaging (MRI) involving the cerebral hemispheres, brainstem, cerebellum, or spinal cord.^[1]^ Although ADEM is classically confined to the central nervous system (CNS), peripheral nervous system (PNS) involvement has been increasingly recognized in recent studies, with electrophysiological abnormalities such as neuropathy reported in a subset of adult patients.^[2]^ Combined central and peripheral demyelination reflects a broader immunopathological spectrum and presents diagnostic and therapeutic challenges due to overlapping features with other demyelinating disorders and atypical clinical courses.^[2]^ We report a rare case of ADEM in a young adult male with prominent pure motor axonal peripheral neuropathy, emphasizing distinct clinical, electrophysiological, and radiological features that distinguish it from classical presentations.

## 2. Case presentation

A 22-year-old Asian male, previously healthy with no significant past medical history, presented with an acute encephalopathic illness. Five days prior to presentation, he had experienced a self-limiting upper respiratory tract infection characterized by sore throat, cough, and low-grade fever, with no history of recent vaccination. He subsequently developed a mild, diffuse headache followed within 24 hours by a single episode of generalized tonic–clonic seizure lasting approximately 2 minutes, associated with post-ictal confusion and progressive alteration in sensorium. There was no history of preceding trauma, toxin exposure, substance abuse, or hypoglycemic episodes. There was no history of vomiting, diarrhea, ear discharge, rash, visual disturbances, bowel or bladder dysfunction, or recent travel. He had no prior psychiatric illness, autoimmune disease, demyelinating disorder, seizure disorder, or relevant family history.

### 2.1. Examination findings

On admission, the patient was drowsy with a Glasgow Coma Scale score of E2V2M5. He opened his eyes only to nociceptive stimuli and did not follow commands or communicate meaningfully. Pupils were equal and reactive, and fundoscopic examination revealed no papilledema. Cranial nerve examination did not reveal any focal deficits. Motor examination showed preserved localization and antigravity movements in the upper limbs, while there was complete absence of voluntary movement in both lower limbs (Medical Research Council grade 0/5). Muscle tone was reduced in the lower limbs. Deep tendon reflexes were diminished in the lower limbs and preserved in the upper limbs, with equivocal plantar responses bilaterally. Sensory examination was initially limited due to impaired consciousness, and there were no clinical signs of meningeal irritation. The patient was unconscious so he was catheterized on day 1, but there was no episode of fecal incontinence. The remainder of the systemic examination was unremarkable.

### 2.2. Initial workup and management

In view of the acute onset of seizures and encephalopathy, a provisional diagnosis of acute encephalitis was made, with differentials including viral encephalitis, autoimmune encephalitis, and acute demyelinating encephalomyelitis. A non-contrast computed tomography scan of the brain was unremarkable. Cerebrospinal fluid (CSF) analysis revealed mild lymphocytic pleocytosis with white blood cells 12/mm^3^, mildly elevated protein level of 60 mg/dL, and normal glucose concentration. CSF Gram stain, culture, and viral and bacterial polymerase chain reaction studies were negative. Empirical treatment with intravenous acyclovir and ceftriaxone was initiated, along with dexamethasone. Due to worsening consciousness, the patient was intubated for airway protection and catheterized for supportive care.

### 2.3. Neuroimaging and diagnosis

**Figure 1.**
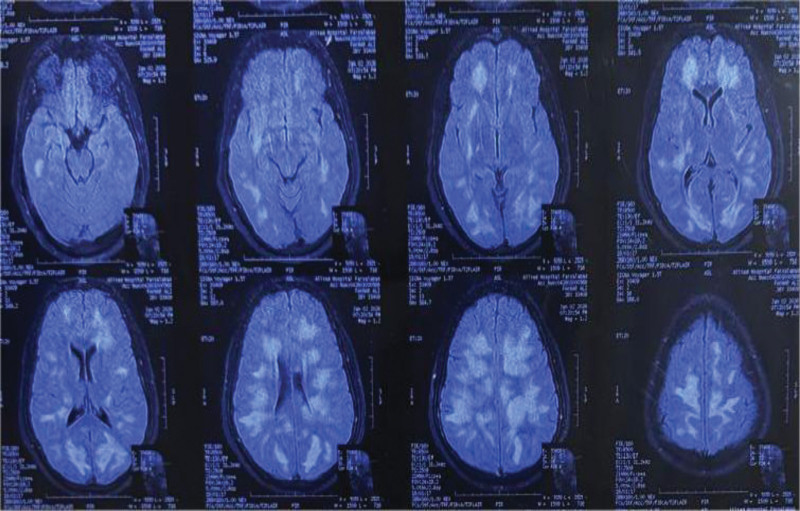
Magnetic resonance imaging of the brain demonstrating multiple asymmetric, poorly marginated hyperintense lesions involving the bilateral supratentorial white matter on FLAIR sequences, without significant diffusion restriction or contrast enhancement, consistent with an acute demyelinating process suggestive of acute demyelinating encephalomyelitis. FLAIR = fluid-attenuated inversion recovery.

Subsequent MRI of the brain demonstrated multiple asymmetric, poorly marginated hyperintense lesions on T2-weighted and fluid-attenuated inversion recovery sequences involving the bilateral supratentorial white matter, without significant diffusion restriction or contrast enhancement (Fig. 1). The distribution and morphology of the lesions were characteristic of a demyelinating process, leading to a diagnosis of acute demyelinating encephalomyelitis, and antimicrobial therapy was discontinued. High-dose intravenous methylprednisolone pulse therapy was administered for 5 days.

### 2.4. Clinical course and further evaluation

Following treatment, the patient showed marked improvement in consciousness, became fully alert, followed verbal commands, and was able to communicate. Upper limb strength improved significantly; however, he continued to have flaccid paraplegia of both lower limbs. On reexamination, superficial and deep sensations were intact in all limbs, and lower limb reflexes remained reduced. In view of the persistent lower limb weakness, spinal cord involvement was considered; however, MRI of the cervical and thoracic spine was normal, with no evidence of myelitis.

### 2.5. PNS involvement

**Figure 2.**
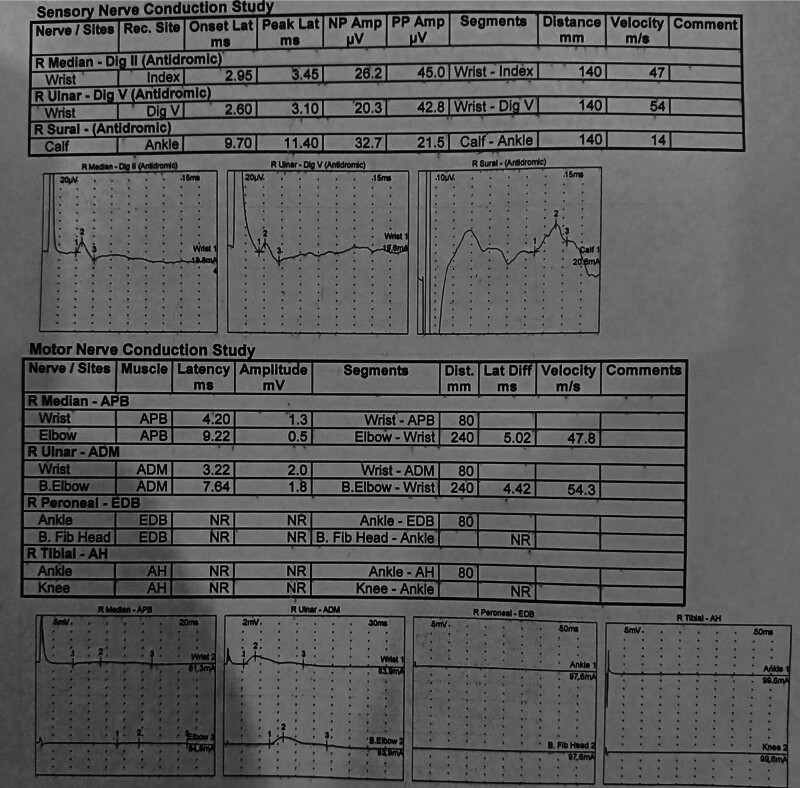
Nerve conduction studies showing a predominantly motor axonal neuropathy affecting the lower limbs, characterized by no response of compound muscle action potential amplitudes and relative sparing of upper limb nerves with preserved sensory nerve action potentials but reduced velocity in lower limb nerves indicating demyelinating findings are also present.

Nerve conduction studies showed a predominantly motor axonal neuropathy affecting the lower limbs, characterized by no response of compound muscle action potential amplitudes and relative sparing of upper limb nerves with preserved sensory nerve action potentials but reduced velocity in lower limb nerves indicating demyelinating findings are also present (Fig. 2). These findings suggested PNS involvement associated with acute demyelinating encephalomyelitis. Testing for autoimmune encephalitis antibodies – including N-methyl-D-aspartate receptor antibody, leucine-rich glioma-inactivated 1 antibody, contactin-associated protein-like 2 antibody, α-amino-3-hydroxy-5-methyl-4-isoxazolepropionic acid receptor antibody, and gamma-aminobutyric acid receptor antibodies – in both serum and CSF, as well as evaluation for secondary CNS vasculitis (antinuclear antibody, cytoplasmic anti-neutrophil cytoplasmic antibody, perinuclear anti-neutrophil cytoplasmic antibody) and assessment for peripheral nerve involvement (anti-ganglioside antibodies and NF-155 antibody), were all negative. Testing for central demyelinating conditions, including oligoclonal bands, myelin oligodendrocyte glycoprotein antibody, and aquaporin-IV, was negative.

### 2.6. Further management and outcome

**Figure 3.**
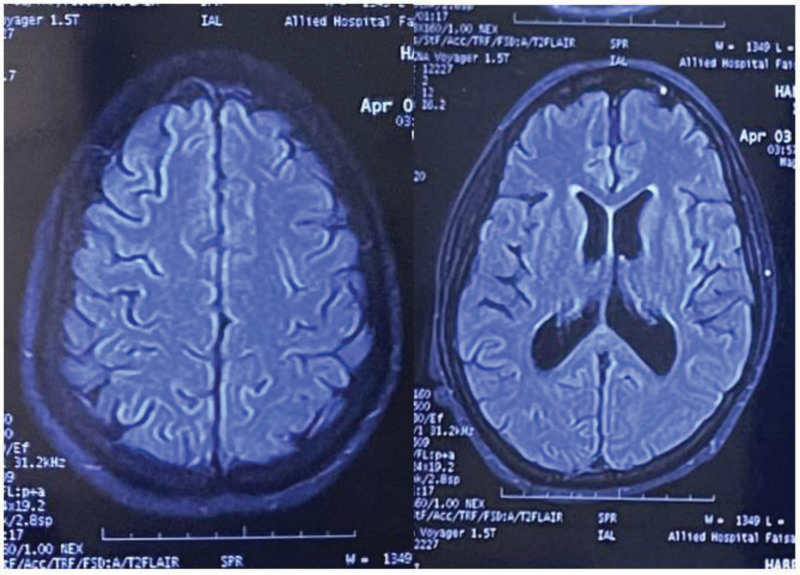
Follow-up magnetic resonance imaging of the brain performed 3 months after treatment demonstrating complete resolution of previously noted supratentorial white matter lesions, supporting the diagnosis of monophasic acute demyelinating encephalomyelitis.

Given the suboptimal motor recovery following corticosteroid therapy, therapeutic plasma exchange was initiated. After 5 sessions of plasma exchange, along with intensive physiotherapy, gradual improvement in lower limb strength was observed. By 2 weeks, the patient was able to stand and ambulate with mild support. A repeat MRI of the brain performed 3 weeks later showed complete resolution of the previously noted white matter lesions, further supporting the diagnosis of acute demyelinating encephalomyelitis. At follow-up 3 months later, the patient demonstrated near-complete neurological recovery, and repeat neuroimaging remained normal (Fig. 3).

In conclusion, this case highlights a rare presentation of acute demyelinating encephalomyelitis with PNS involvement in a young adult. Unlike most previously reported cases, which predominantly involve older patients and demyelinating peripheral neuropathy, our patient was younger in age and exhibited a predominantly motor, axonal pattern of peripheral neuropathy with sparing of sensory fibers. The combination of early CNS involvement, pure motor axonal neuropathy, and favorable response to plasma exchange represents a distinctive clinical phenotype. This case emphasizes the importance of considering PNS involvement in acute demyelinating encephalomyelitis, particularly in younger patients with persistent weakness despite appropriate immunotherapy, as timely recognition may have significant therapeutic and prognostic implications.

## 3. Discussion

ADEM is a rare, monophasic, autoimmune inflammatory disorder predominantly affecting the CNS and typically occurring after an infectious illness or, less commonly, vaccination.^[3,4]^ It characteristically presents with acute encephalopathy and multifocal neurologic deficits, with MRI demonstrating widespread, asymmetric, poorly marginated white matter lesions.^[3,4]^ CSF analysis often reveals mild lymphocytic pleocytosis and elevated protein, but oligoclonal bands are infrequent.^[3]^ While ADEM is more common in children and generally has a favorable prognosis following immunotherapy, adults may have a more severe clinical course and poorer outcomes.^[3,5]^ PNS involvement in ADEM is recognized but uncommon; when present, it may manifest with clinical or neurophysiological evidence of neuropathy and has been associated with worse recovery.^[5,6]^

The coexistence of CNS demyelination with concurrent or subsequent PNS involvement, sometimes referred to as combined central and peripheral demyelination, suggests a shared or overlapping autoimmune process targeting both central and peripheral myelin. Although classical ADEM primarily involves CNS white matter, several case reports have documented combined involvement in adults, including demyelinating polyradiculoneuropathy and electrophysiological evidence of peripheral demyelination.^[6,7]^ These manifestations highlight that, in some individuals, pathogenic immune responses may extend beyond oligodendrocyte targets to include peripheral myelin or axonal structures. In contrast to most reported cases, which involve demyelinating peripheral changes, our patient exhibited predominantly axonal motor neuropathy on nerve conduction studies while sensory nerve function remained intact – a pattern more consistent with acute motor axonal neuropathy, a subtype of peripheral involvement rarely described in ADEM.^[4,5,7]^ This underscores the heterogeneity of peripheral involvement in ADEM and suggests a broader pathophysiological spectrum that may include axon-targeted immune injury.

Another distinguishing feature of this case is the patient’s young age. Although ADEM typically affects children and young adults, combined central and peripheral involvement has most often been reported in older adults in the limited literature.^[5,6]^ The presence of severe motor axonal neuropathy in a young adult suggests that age should not preclude consideration of peripheral involvement and that the immunopathogenic mechanisms in these atypical presentations may differ from classic central-only ADEM.

MRI findings in our case were typical for ADEM, with asymmetric supratentorial white matter hyperintensities, supporting the primary diagnosis in the context of postinfectious encephalopathy.^[3]^ The disappearance of these lesions on follow-up imaging after appropriate immunomodulatory treatment further confirms the monophasic nature of the disorder and is consistent with expected radiological resolution in classical ADEM.^[3]^ The favorable response to plasma exchange after corticosteroid therapy also aligns with current practice, as second-line immunotherapies are recommended for patients with incomplete response to steroids.^[3,5]^

A landmark nationwide survey from Japan published in *JNNP* in 2016 emphasized several important features of CCPD: it often presents with T2 hyperintense CNS lesions on MRI and electrophysiological evidence of PNS demyelination, fulfilling diagnostic criteria for both CNS and PNS involvement. In this cohort, symptoms such as sensory disturbance, motor weakness, and gait impairment were common, and abnormally elevated CSF protein levels were frequently observed, although oligoclonal immunoglobulin G bands were less common than in isolated CNS demyelinating diseases.^[8]^

Given the rarity of significant peripheral motor axonal involvement in ADEM, clinicians should maintain a high index of suspicion for PNS involvement in patients presenting with disproportionate weakness or neurophysiological abnormalities, even in younger patients.^[5,6]^ Electrophysiological studies are essential for detecting subclinical or overt peripheral nerve pathology and can influence management decisions, such as the early use of therapeutic plasma exchange or intravenous immunoglobulin.

In summary, this case illustrates a rare variant of ADEM with combined central and peripheral involvement in a young adult, characterized by predominant motor axonal neuropathy. Recognition of this atypical presentation broadens the clinical spectrum of ADEM and emphasizes the importance of comprehensive neurodiagnostic evaluation and individualized immunotherapy to optimize outcomes.

## Author contributions

**Validation:** Fiza Nisar.

**Investigation:** Danish Yousuf.

**Software:** Gohar Mushtaq.

**Data curation:** Muhammad Abdullah Ali.

**Conceptualization:** Anousha Tanveer.

**Visualization:** Hammad Azam, Mukesh Kumar Sharma.

**Writing – original draft:** Aasim Ali, Muhammad Asad Shabbir.

**Writing – review & editing:** Muhammad Shamoon.
